# Biodegradation of lignin monomers and bioconversion of ferulic acid to vanillic acid by *Paraburkholderia aromaticivorans* AR20-38 isolated from Alpine forest soil

**DOI:** 10.1007/s00253-021-11215-z

**Published:** 2021-03-09

**Authors:** Rosa Margesin, Georg Volgger, Andreas O. Wagner, Dechao Zhang, Caroline Poyntner

**Affiliations:** 1 Institute of Microbiology, University of Innsbruck, Technikerstrasse 25, 6020 Innsbruck, Austria; 2 Institute of Oceanology, Chinese Academy of Sciences, Qingdao, 266071 China; 3 University of Chinese Academy of Sciences, Beijing, 100049 China

**Keywords:** Lignin, Ferulic acid, Vanillic acid, Bioconversion, Cold-adapted ligninolytic *Paraburkholderia*

## Abstract

**Abstract:**

Lignin bio-valorization is an emerging field of applied biotechnology and has not yet been studied at low temperatures. *Paraburkholderia aromaticivorans* AR20-38 was examined for its potential to degrade six selected lignin monomers (syringic acid, p-coumaric acid, 4-hydroxybenzoic acid, ferulic acid, vanillic acid, benzoic acid) from different upper funneling aromatic pathways. The strain degraded four of these compounds at 10°C, 20°C, and 30°C; syringic acid and vanillic acid were not utilized as sole carbon source. The degradation of 5 mM and 10 mM ferulic acid was accompanied by the stable accumulation of high amounts of the value-added product vanillic acid (85–89% molar yield; 760 and 1540 mg l^−1^, respectively) over the whole temperature range tested. The presence of essential genes required for reactions in the upper funneling pathways was confirmed in the genome. This is the first report on biodegradation of lignin monomers and the stable vanillic acid production at low and moderate temperatures by *P. aromaticivorans*.

**Key points:**

• *Paraburkholderia aromaticivorans AR20-38 successfully degrades four lignin monomers.*

• *Successful degradation study at low (10°C) and moderate temperatures (20–30°C).*

• *Biotechnological value: high yield of vanillic acid produced from ferulic acid.*

## Introduction

Natural lignin is the second most abundant organic substance in the world (next to cellulose) (Ľudmila et al. 2015; Ganewatta et al. 2019). The global amount of available lignin in the biosphere is approximately 300 billion tons, with an annual increase of approximately 20 billion tons (Becker and Wittmann 2019). It is found in the secondary cell wall of lignocellulosic plants and provides structural strength, impermeability, water transport in the cell wall, and protection from pathogens (Brink et al. 2019). Natural lignin is a complex aromatic heteropolymer and consists of a matrix of aromatic (phenolic) and aliphatic substances. Its complex three-dimensional structure is a result of the polymerization of three phenylpropane units that originate from the three aromatic alcohols p-coumaryl, conifery, and sinapyl (Liu et al. 2018; Ganewatta et al. 2019).

Due to its complex and heterogeneous composition and structure, natural lignin is one of the most recalcitrant biopolymers (Bugg et al. 2011; Ganewatta et al. 2019). However, it does not accumulate in nature. After plant death, natural lignin undergoes natural biodegradation by soil microorganisms, which results in the formation of soil organic matter. Remarkable ligninolytic activity has been reported from a number of microorganisms. Studies on lignin biodegradation have focused primarily on basidiomycetous fungi (white rot and brown rot) since the mid-1980s. However, no commercial biocatalytic process for lignin depolymerization could be developed so far by using fungal isolates, which can be attributed to the difficulties associated with fungal genetic manipulation and fungal protein expression (Bugg et al. 2011). Ligninolytic bacteria have numerous advantages over fungi for biotechnological applications, such as rapid growth, easy genetic manipulation due to the small genome size, high stability and resilience in varying environmental conditions, and a great flexibility in the metabolism of lignin-related aromatic compounds (Bugg et al. 2011; Wang et al. 2018; Brink et al. 2019). Culturable lignin-degrading bacteria belong mostly to the phyla *Actinobacteria*, *Firmicutes*, and *Proteobacteria*. The classes *Alpha* and *Gammaproteobacteria* dominate among *Proteobacteria* (Bugg et al. 2011; Tian et al. 2014; Brink et al. 2019), while reports on the utilization of lignin by *Betaproteobacteria* are limited (Morya et al. 2019).

Currently, technical lignin is produced worldwide in high amounts in the pulp and paper industry and mainly used as an energy source to generate process steam and electricity. Technical lignin is also used in new biorefinery concepts, including 2nd-generation ethanol, and represents a major renewable source of aromatic and phenolic bio-products, which would be valuable raw materials for the synthesis of fine chemicals and materials and for the food and flavor industry (Bugg et al. 2011; Palazzolo and Kurina-Sanz 2016). In this context, there has been growing interest in the use of ferulic acid (FA) as feedstock for biocatalytic conversion into other value-added products, such as vanillin and vanillic acid (VA) (Rosazza et al. 1995). The study of ligninolytic microorganisms and the assessment of their potential is essential for lignin valorization (Ravi et al. 2018). More studies are needed to expand our knowledge on lignin monomer degradation (Wang et al. 2018).

Studies on lignin-degrading microorganisms have been performed under mesophilic conditions; however, ligninolytic activity under low-temperature conditions has not yet been studied. Cold-adapted microorganisms play a key ecological role in their natural habitats for nutrient cycling, litter degradation, and many other processes (Margesin and Collins 2019). Low-temperature biodegradation of a broad range of organic compounds, including alkanes and aromatic and polyaromatic hydrocarbons, has been reported in a number of studies (e.g., Bej et al. 2010; Margesin et al. 2013). Although low temperature results in a lower conversion rate, microbial activity under cold conditions offers a number of advantages for biotechnological processes and is of particular interest for low-energy treatments (Collins and Margesin 2019), e.g., for bioremediation in cold conditions. Furthermore, understanding lignin degradation in low-temperature areas is of high ecological importance.

In an earlier study (Berger et al. 2021), we demonstrated the ability of a high number of bacterial strains isolated from soil from an Alpine coniferous forest site to utilize both water-soluble (leaf-soluble sugar (LSS)) and water-insoluble (lignin alkali) forms of lignin as sole carbon source. Among these strains, *Paraburkholderia aromaticivorans* AR20-38 was characterized by its ability to utilize high amounts of LSS and phenol as sole carbon source over its whole growth temperature range on these compounds (0–30°C) and was therefore selected for further studies on lignin monomer degradation. It was the aim of this study to assess the ability of *P. aromaticivorans* AR20-38 (a member of *Betaproteobacteria*) to degrade six representative lignin monomers (syringic acid, p-coumaric acid, 4-hydroxybenzoic acid, ferulic acid, vanillic acid, benzoic acid) that represent different branches of the upper funneling catabolic pathways for the bacterial metabolism of lignin components (sinapyl, p-coumaryl, coniferyl, benzoyl; Brink et al. 2019). Biodegradation studies were performed over a broad temperature range (10–30°C). Special attention was paid to the bioconversion of FA to VA, and the sequenced draft genome (Poyntner et al. 2020) was analyzed for the presence of known genes being essential for the upper funneling aromatic pathways. Here, we report for the first time the degradation potential for lignin model compounds and the stable production of VA from FA under low-temperature conditions.

## Materials and methods

### Strain

The bacterial strain used in this study was isolated from soil from an Alpine coniferous forest site located 7 km north of Bozen/Bolzano, Italy, below the Rittner Horn at an altitude of 1724–1737 m above sea level as described (França et al. 2016) and identified as member of the species *P. aromaticivorans* (GenBank accession no. MT281269; Berger et al. 2021). The whole genome was sequenced, and the resulting draft genome sequence of the strain has been recently described (Poyntner et al. 2020) (GenBank BioProject number PRJNA624061). The strain was deposited in the China General Microbiological Culture Collection center under the number CGMCC 1.18749 and is publicly available. The strain was stored at −80°C using ROTI©Store cryovials.

### Chemicals

Syringic acid (SA; Alfa Aesar 5003), p-coumaric acid (CA; Sigma C9008), 4-hydroxybenzoic acid (HBA; Serva 25271), trans-ferulic acid (FA; Sigma-Aldrich 128708), vanillic acid (VA; Merck 841025), and benzoic acid (BA; Sigma-Aldrich 242381) were of chromatographic pure grade. Stock solutions (0.5 M) were prepared in DMSO and stored at 4°C. Preliminary studies showed that the amount of DMSO added to the cultivation flasks by the addition of these compounds did not affect bacterial growth.

### Biodegradation of lignin monomers (model compounds)

The biodegradation assays were carried out in 100-ml Erlenmeyer flasks with screw caps containing 20 ml of mineral salts medium (MM) supplemented with 5 mM (final concentration) of one of the target compounds (SA, CA, HBA, FA, VA, BA) as sole carbon source. To ensure sufficient aeration, the culture flasks were opened regularly for sampling under sterile conditions. The biodegradation of FA was also evaluated with a final concentration of 10 mM FA, using a 1 M stock solution. The pH-neutral MM contained (compositions indicated per liter) Na_2_HPO_4_ x 2H_2_O (3.5 g), KH_2_PO_4_ (2 g), (NH_4_)_2_SO_4_ (1 g), MgSO_4_ x 7 H_2_O (0.2 g), Ca(NO_3_)_2_ x 4 H_2_O (0.05 g), ammonium iron(III) citrate (10 mg), a trace element, and a vitamin solution (Schlegel 1992; Margesin and Schinner 1997). The pH of the medium was adjusted to 7.0 after the addition of the compounds. For inoculation, a preculture prepared in MM containing glucose (2 g l^−1^) as carbon source was prepared. The bacterial cells were separated by centrifugation (10,000 x g for 10 min), washed twice with sterile MM, and suspended in MM. The initial (t0) optical density at 600 nm (OD600) in the inoculated flasks was adjusted to 0.05. Two negative controls contained (1) sterile medium supplemented with lignin monomers and (2) inoculated medium without the target compounds. The flasks were incubated in triplicate at 10°C, 20°C, and 30°C and 150 rpm. Growth (OD600), pH of the cultures, and the concentration of the lignin monomers were monitored in samples collected at regular time intervals.

### HPLC analysis

Lignin monomers were quantified by using HPLC analysis. The samples for the analysis were centrifuged for 10 min at 20,000 x g to remove all larger particles. The supernatants were frozen at −20°C before HPLC analysis. At least 0.7 mL of the supernatant was filtered through a 0.2-μm RC filter. The analysis was performed on a Shimadzu Prominence system via a RFQ Fast Acid column (50 x 7.8 mm, Phenomenex, Germany) at 70°C. A time program starting with a flow rate of 0.25 ml min^−1^ for 20 min, then ramping to 1.0 ml min^−1^ within 10 min, and finally keeping this flow rate until method stop was used with 5 mM sulfuric acid as the mobile phase. The separated components were measured via a UV detector at 220 nm and crosschecked at 270 nm. As external standards, SA, CA, HBA, FA, VA, and BA were injected in concentrations of 1, 5, and 10 mM to obtain a calibration curve.

### Genome analysis

The draft genome of *P. aromaticivorans* AR20-38 (Poyntner et al. 2020) was analyzed for the presence of genes involved in upper lignin funneling pathways according to the eLignin Microbial Database (www.elignindatabase.com; Ravi et al. 2018;  Brink et al. 2019) and genomes of comparable strains (Lee et al. 2019; Morya et al. 2019). Genes coding for essential enzymes in the bacterial lignin metabolism were identified in the annotated genome through the databases SwissProt (Bairoch and Apweiler 2000), COG (Galperin et al. 2015), TCDB (Saier et al. 2014), GO (Ashburner et al. 2000; The Gene Ontology Consortium 2019), PHI (Winnenburg et al.^ 2008^; Urban et al. 2020), VFDB (Chen et al. 2012), CARD (Alcock et al. 2020), Effective T3 (Arnold et al. 2009), CAZy (Cantarel et al. 2009) RefSeq Non-Redundant Protein Database (O'Leary et al. 2016), and Pfam (El-Gebali et al. 2019).

## Results

### Biodegradation of lignin monomers

No abiotic losses of lignin monomers were detected at any of the incubation temperatures over the whole incubation periods (data not shown). The difference between the initial and the residual concentration of lignin monomers could thus be attributed to biodegradation. The initial pH of 7.0 in the culture media was not affected during growth and biodegradation.

Strain *P. aromaticivorans* AR20-38 was able to utilize four out of the six tested lignin monomers (CA, HBA, FA, BA) as sole carbon and energy source at all three test temperatures (10, 20, and 30°C). SA and VA could not be utilized at any of the temperatures tested.

**Fig. 1:**
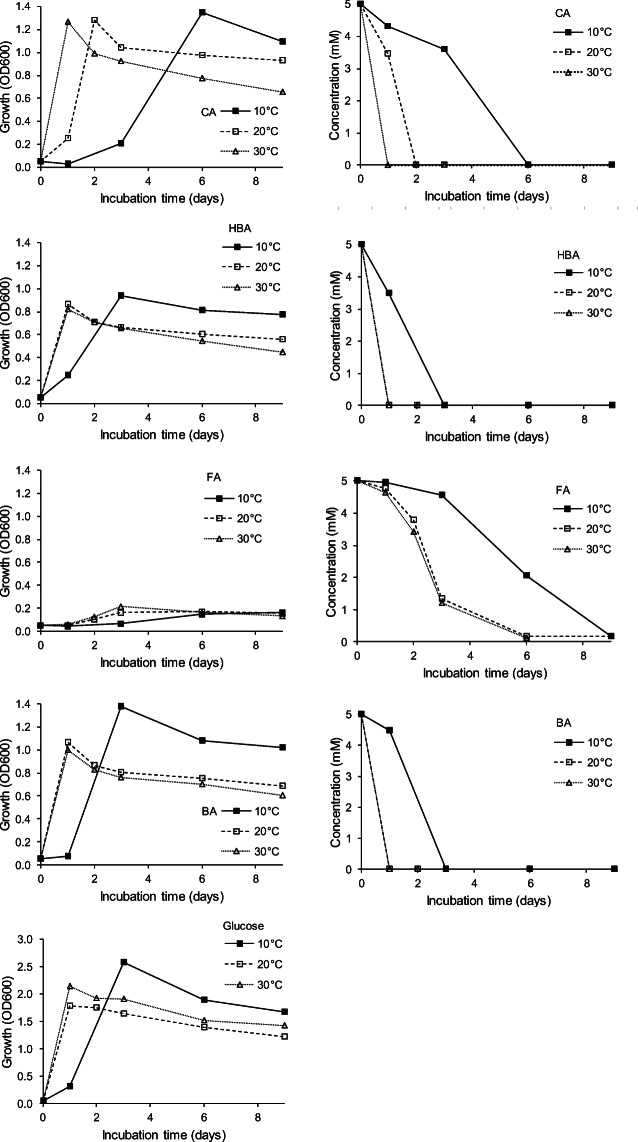
Effect of temperature on growth (left panels) and biodegradation (right panels) of lignin model compounds (CA, p-coumaric acid; HBA, 4-hydroxybenzoic acid; FA, trans-ferulic acid; BA, benzoic acid) as sole carbon source by *Paraburkholderia aromaticivorans* AR20-38 (mean values of three replicates; SDs were ≤10%). VA production during FA consumption is not shown (see Fig. 2). The effect of temperature on growth with glucose as sole carbon source is shown in the left panel on the bottom

**Fig. 2:**
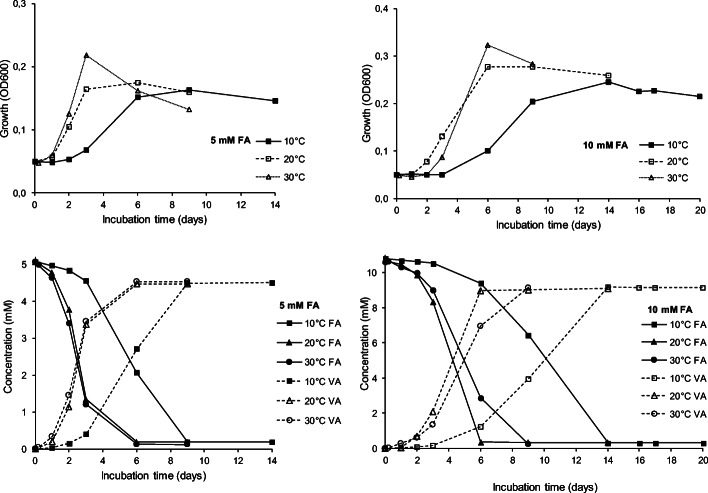
Effect of temperature (10°C, 20°C, 30°C) and FA concentration (left panels: 5 mM; right panels: 10 mM) on growth (upper panels) and bioconversion of FA (bottom panels) by *Paraburkholderia aromaticivorans* AR20-38 (mean values of three replicates; SDs were <5 %)

An increase in temperature resulted in accelerated growth and degradation (Fig. 1). At a cultivation temperature of 10°C, 5 mM HBA and BA were fully degraded after 3 days, while 6 and 9 days were needed for the full degradation of 5 mM CA and FA, respectively. At 20°C, the four compounds were undetectable after 1 (HBA, BA), 2 (CA), and 6 (FA) days, respectively. At 30°C, CA, HBA, and BA were consumed after 1 day, while 6 days were needed for FA. The degradation of FA was accompanied with the stable accumulation of VA due to bioconversion. Independent of the FA concentration or the incubation temperature, the maximum of VA was released when FA was fully degraded (see also below, Fig. 2).

The substrate preference among the lignin model compounds tested in this study was clearly visible at 10°C (HBA ≥ BA > CA > FA); at higher temperatures, no distinction between the degradation performance of HBA and BA (20°C) or HBA, BA, and CA (30°C) was detectable.

The degradation of the lignin monomers paralleled growth (OD600); growth declined as soon as the carbon source was depleted (as also observed by Ravi et al. 2017). OD600 values, however, were not indicative of the degradation capacity. Biomass production was significantly higher during the degradation of CA (OD600 = 1.3–1.4), BA (OD600 = 1–1.4), and HBA (OD600 = 0.8–0.9) (Fig. 1) than during FA degradation (OD600 = 0.2) (Fig. 2). This occurred independent of the incubation temperature.

In all cases, growth at 10°C was delayed compared to growth at 20°C and 30°C; however, it resulted in significantly higher biomass production in case of BA and glucose as sole carbon source. A tendency of increased biomass production at 10°C, although not significant, was also visible for the other compounds consumed by the strain. Growth with glucose as the sole carbon source resulted in higher biomass production (OD600 = 1.8–2.6) compared to growth with lignin monomers (Fig. 1).

### Bioconversion of trans-ferulic acid to vanillic acid

The degradation of FA was accompanied by the production of VA. This bioconversion occurred independently of the initial concentration of FA (5 mM, 10 mM) or the incubation temperature (10, 20, 30°C) (Fig. 2). When FA was provided as sole carbon source, almost 75% of the initial concentration of 5 mM FA were degraded after 3 days at 20 and 30°C (not detectable after 6 days), while still 40% were found after 6 days at 10°C (no detection after 9 days). An increase in the initial FA concentration (10 mM) resulted in faster degradation at 20°C than at 30°C (not detectable after 6 and 9 days, respectively) or 10°C (not detectable after 14 days). Thus, a delay of the period needed for full degradation was only detected at 10°C and 30°C, however, not at 20°C. This points to a temperature of 20°C as the optimum temperature in terms of the degradation of high amounts of FA.

Using 5 mM (1.022 g l^−1^) FA as the sole source or carbon and energy, a maximum amount of 0.76 mg VA l^−1^ (molar yield 88–89%) was obtained in the medium at all three incubation temperatures. When the target concentration of 10 mM FA (10.6–10.8 mM) was provided, the molar yield (85–86%; 1.5 g l^−1^) was only slightly lower (Table 1).

**Table 1:** Effect of temperature (10°C, 20°C, 30°C) on VA production from 5 mM and 10 mM FA obtained with *Paraburkholderia aromaticivorans* AR20-38 (mean values of three replicates ± SD)

Temperature	FA concentration (t0)		VA production				
	mM	mg l^−1^	mM	mg l^−1^	g (l x OD600) ^−1^	Molar yield (%)	Mass yield (%)
10°C	5.1 ± 0.15	984 ± 29	4.5 ± 0.02	757 ± 3	4.6 ± 0.02	88.9 ± 2.42	77.0 ± 2.10
20°C	5.1 ± 0.15	984 ± 29	4.5 ± 0.02	752 ± 4	4.3 ± 0.02	88.3 ± 2.46	76.5 ± 2.13
30°C	5.1 ± 0.15	984 ± 29	4.5 ± 0.03	760 ± 4	3.5 ± 0.02	89.3 ± 2.44	77.3 ± 2.11
10°C	10.8 ± 0.04	2098 ± 8	9.2 ± 0.03	1541 ± 5	6.3 ± 0.02	84.8 ± 0.39	73.4 ± 0.33
20°C	10.7 ± 0.17	2076 ± 32	9.1 ± 0.04	1531 ± 7	5.5 ± 0.02	85.1 ± 0.95	73.7 ± 0.82
30°C	10.6 ± 0.23	2063 ± 44	9.1 ± 0.01	1537 ± 2	4.8 ± 0.01	86.1 ± 1.76	74.6 ± 1.53

### Genome analysis

The analysis of the three chromosomes and plasmid of the draft genome of *P. aromaticivorans* AR20-38 (Poyntner et al. 2020) revealed the presence of various genes involved in the degradation of the studied lignin model compounds (see Table 2 for details and loci). All genes related to lignin degradation were found in the core genome on chromosome 1, 2, and 3.

**Table 2:** Annotated genes of the upper funneling pathway based on the eLignin database (Brink et al. 2019) and potential genes involved in lignin degradation

Gene ID	Database	Database no.	Locus	Pathway	*E*-value
Genes involved in the funneling pathway lignin degradation (eLignin Database)					
Vanillin dehydrogenase	SwissProt	O69763	Chromosome 2	Vanillin to vanillic acid	2.3e-196
Feruloyl-CoA synthase	PHI	PHI:341Z, Q8XT88	Chromosome 1 and 2	Ferulic acid to feruloyl-CoA	1.2e-112
Enoyl-CoA Hydratase/lyase	SwissProt	Q2RQ36	Chromosome 1	p-Coumaroyl-CoA to 4-hydroxybenzaldehyde	1.4e-36
Hydroxybenzoate 3-Monooxygenase	SwissProt, Non-Redundant Protein Database	Q6SSJ6, WP_012433227.1	Chromosome 1, 2, and 3	4-Hydroxybenzoic acid to protocatechuic acid	7.0e-214; 2.6e-221
Benzoate 1, 2-dioxygenase	Non-Redundant Protein Database	WP_012432614.1	Chromosome 1 and 3	Benzoic acid to 3,5-cyclohexadiene-1,2-diol-1-carboxylate	6.1e-267
1,6-Dihydroxycyclohexa-2,4-diene-1-carboxylate dehydrogenase	SwissProt	P07772	Chromosome 1 and 3	3,5-Cyclohexadiene-1,2-diol-1-carboxylate to catechol	9.6e-77
Catechol 1,2-dioxygenase	SwissProt	O33950	Chromosome 3	Catechol to cis,cis-muconic acid	2.1e-144
Catechol 2,3-dioxygenase	COG, Non-Redundant Protein Database	YP_559607, YP_00135324, YP_003280458, YP_004682700, YP_560354, WP_062170910.1	Chromosome 3	Catechol + O2 → (2Z,4E)-2-hydroxy-6-oxohexa-2,4-dienoate	7.8e-68; 3.0e-45; 5.7e-65; 1.5e-109;4.0e-64; 4.3e-155
Potential genes involved in lignin degradation					
Vanillate O-demethylase oxidoreductase	Non-Redundant Protein Database SwissProt	WP_035937771.1, WP_063500164.1 O05617	Chromosome 1 and 3	Vanillic acid to catecholic structures, protocatechuate and gallate (Kamimura et al. 2017)	2.1e-154; 1.3e-165; 4.1e-93
Enoyl-CoA Hydratase/isomerase	COG, Non-Redundant Protein Database, SwissProt	YP_001413905, YP_002798614, YP_554216, YP_554155, YP_556021, YP_555851, YP_004680815, YP_005026757, YP_559584, WP_005793322.1, WP_028231531.1, WP_035521646.1, WP_011492940.1, WP_012426569.1, WP_012427501.1, WP_012404161.1, WP_030102796.1, WP_028194277.1, WP_069266866.1, WP_064271658.1, WP_012431551.1, WP_012431843.1, WP_035551717.1, WP_012432712.1, WP_035997457.1, WP_011489469.1, WP_011489931.1, WP_012434439.1, SDH36466.1, SDB90530.1, SAL69706.1, SAL32406.1, G4V4T7, O34893	Chromosome 1, 2, and 3	Feruloyl-SCoA to vanillin and acetyl-SCoA (Gasson et al. 1998)	5.0e-66; 1.0e-130; 1.6e-201; 1.7e-163; 1.1e-58; 3.5e-123; 2.6e-135; 7.1e-52; 0; 2.4e-130; 1.5e-132; 4.0e-138; 4.2e-157; 1.6e-139; 9.6e-143; 3.7e-131; 4.6e-137; 9.5e-154; 2.4e-98; 1.0e-139; 5.7e-165; 1.4e-138; 4.6e-151; 7.8e-131; 1.3e-136; 4.7e-139; 3.4e-137; 3.5e-134; 2.5e-116; 2.8e-123; 4.3e-116; 6.7e-54; 2.5e-46
Benzaldehyde dehydrogenase	Non-Redundant Protein Database	WP_028221099.1	Chromosome 3	Syringyl lignin catabolism (Kamimura et al. 2017)	2.7e-236
Benzoyl-CoA oxygenase/reductase	Non-Redundant Protein Database	WP_012433680.1	Chromosome 3	Putative genes assisting lignin breakdown (Kumar et al. 2018)	1.2e-240

Regarding CA degradation, genome analysis showed the presence of the gene encoding for enoyl-CA hydratase/lyase, which according to the suggested upper funneling aromatic pathway leads to the production of 4-hydroxybenzaldehyde (eLignin database). Genes related to enzymes involved in 4-hydroxybenzaldehye consumption and thus HBA production were not present. The degradation of HBA, however, was confirmed by the presence of four genes encoding for 4-hydroxybenzoate 3-monooxygenase, which results in the formation of protocatechuate.

FA degradation resulted in the synthesis and stable accumulation of VA. The transient production of the intermediate vanillin during FA bioconversion was evidenced by the presence of two genes encoding for the enzyme involved in the vanillin production from FA (feruloyl-CoA synthase). Genes for the enzyme involved in feruloyl-CA degradation (trans-feruloyl-CoA hydratase) were not found. The degradation of vanillin to VA was evidenced by the presence of a gene encoding for vanillin dehydrogenase (vanillin I funneling pathway; eLignin database). The stability of this product was confirmed by the inability of the strain to utilize VA as sole carbon source and by the absence of the gene for vanillate monooxygenase, which would produce protocatechuate from VA (vanillate monooxygenase). The presence of vanillate-O-demethylate oxidoreductase indicates the ability for the degradation of VA to catecholic structures, protocatechuate, and gallate (Kamimura et al. 2017), which is in contrast to the biodegradation results.

Syringate O-demethylase, an enzyme responsible for SA degradation to 3-O-methylgallate and 5-methyltetrahydrofolate, was not present in the annotated genome data. In contrast, the enzymes benzoate 1,2-dioxygenase and 1,6-dihydroxycyclohexa-2,4-diene-1-carboxylate-dehydrogenase, essential for BA degradation and leading to 3,5-cyclohexadiene-1,2-diol-1-carboxylate and further to catechol, were present in the genome.

Catechol-1,2-dioxygenase can degrade catechol and is encoded in the annotated genome. It leads to the production of cis,cis-muconic acid. Further, genes for catechol 2,3-dioxygenase were identified, an enzyme known to lead from catechol to (2Z,4E)-2-hydroxy-6-oxohexa-2,4-dienoate. The ability of *P. aromaticivorans* AR20-38 to utilize catechol is evidenced by its ability to utilize catechol as carbon source and to produce the relevant enzyme (Berger et al. 2021). Three additional genes present in the genome are potentially involved in the lignin degradation pathway: enoyl-CoA hydratase/isomerase in the vanillin funneling pathway (Gasson et al. 1998), benzaldehyde dehydrogenase in the syringyl lignin catabolism (Kamimura et al. 2017), and benzoyl-CoA oxygenase/reductase in assisting the breakdown of lignin (Kumar et al. 2018).

In the annotated gene, 980 genes related to transporters were identified (Table 3). In relation to lignin biodegradation, ABC transporters (e.g., vanillin transport), MFS transporters, and RND transporters (Morya et al. 2019) were present. Interestingly, genes encoding the MFS hydroxybenzoate transporters were found 19 times and on all chromosomes.

**Table 3:** Number of transporter and transcriptional regulator genes identified in the annotated genome

Transporter type	Number of annotated genes
All	980
ABC transporter	424
MFS transporter	205
RND transporter	11
Transcriptional regulator	Number of annotated genes
Lys R	232
GntR	110
IclR	121
XRE	48
MarR	122

In the genome, various genes encoding transcriptional regulators (Table 3) are encoded. Similar to *Burkholderia* sp. ISTR5 (Morya et al. 2019), genes belonging to the LysR family were found, known for orthocleavage pathway of catechol. Further genes belonging to GntR, IclR, XRE, and MarR were found, which are known to be involved in hydrocarbon degradation (Morya et al. 2019, Tropel and van der Meer 2004).

## Discussion

In this study, we report the degradation of the four lignin model compounds CA, HBA, FA, and BA and the bioconversion of FA to VA (with stable VA accumulation) over a wide temperature range, including cold conditions (10–30°C), by the bacterial strain *P. aromaticivorans* AR20-38 isolated from soil from an Alpine coniferous forest site. The ability of bacterial strains isolated from this site to utilize lignin as sole carbon source has been reported previously (Berger et al. 2021). Members of the genus *Paraburkholderia* (order *Burkholderiales*, class *Betaproteobacteria*) have been isolated from diverse ecological niches. The majority originates from soils or in association with plant roots (Lee and Jeon 2018; Wilhelm et al. 2020). They are frequently isolated from forest soils (Xiao et al. 2019; Paulitsch et al. 2020; Wilhelm et al. 2020) where they are involved in the decomposition of plant-derived aromatics and appear to play a role as principle contributors to the soil priming effect (Wilhelm et al. 2020). Genome analyses revealed the versatile metabolic capabilities of *Paraburkholderia* representatives, including a member of the species *P. aromaticivorans*, (Lee et al. 2019), for the degradation of organic compounds (Wilhelm et al. 2020 and references therein). *Paraburkholderia* members have been described as degraders of crude oil and aliphatic (n-hexadecane), monoaromatic (benzene; toluene; ethylbenzene; o-, m-, and p-xylene; 4-hydroxybenzoic acid; halogenated phenols), and polycyclic aromatic (naphthalene, phenanthrene) hydrocarbons (Coenye et al. 2004; Li et al. 2017; Yuan et al. 2018; Lee et al. 2019; Wilhelm et al. 2020). However, to the best of our knowledge, no study has previously described the ligninolytic capacity of members of the genus *Paraburkholderia* (1) for biodegradation under low-temperature conditions and (2) for the synthesis of VA from FA. Our study extends knowledge on the role of this genus in lignin utilization and on ligninolytic members of the class *Betaproteobacteria*, which have only rarely been described before (Tian et al. 2014; Brink et al. 2019; Morya et al. 2019).

Our data show that *P. aromaticivorans* AR20-38 was unable to degrade lignin monomers of the sinapyl branch (SA); however, it was able to utilize three of the four main branches of the upper funneling catabolic pathways for the bacterial metabolism of lignin compounds: the p-coumaryl branch (CA and utilization of HA produced from CA), the coniferyl branch (FA, with accumulation of VA), and the benzoyl branch (BA). The preferential degradation of compounds of the p-coumaryl and benzoyl branches (detectable at 10°C) compared to the coniferyl branch (at 10–30°C) was clearly visible and is likely associated to the absence of a methyl group in these structures (BA, CA). Ravi et al. (2017) also observed that 4-hydroxybenzoate was preferentially consumed first, whereas ferulate was always the last consumed substrate by members of the genus *Pseudomonas*.

The genomic analysis supported the findings from biodegradation studies. For the degrading abilities of CA, HBA, FA, and BA, known genes are present in the draft genome. Genes of the upper funneling pathway are encoded in the core genome and are therefore essential for the strain. The genes for catechol degradation are only encoded on the same locus, chromosome 3 (Table 2), and might be regulated similarly, while other genes for the funneling pathway are distributed on two or all chromosomes. Genes encoding transcriptional regulators are found throughout the three chromosomes and are comparable to published (*Para-*)*Burkolderia* genomes (Lee et al. 2019; Morya et al. 2019). Known transcriptional factors for hydrocarbon degradation and catechol metabolism (e.g., LysR family) were identified. The high number of genes in the core genome encoding transporters (Table 3), e.g., ABC transporters, which can be involved in lignin derivative transport (Morya et al. 2019), supports the high biodegradation capability of the studied strain.

In this study, we also demonstrated the stable accumulation of the value-added product VA from FA by *P. aromaticivorans* AR20-38. Interestingly, genes for vanillate-O-demethylate oxidoreductase are present in the genome, which is used to degrade VA to catecholic structures (Kamimura et al. 2017). The strain might down-regulate this gene under the studied conditions and therefore was not able to degrade VA. After complete degradation of FA, no more biomass is produced (Fig. 1). To understand the lack of further VA degradation, detailed experiments are planned. In the genome, several genes, e.g., benzaldehyde dehydrogenase (Table 2), are present, which are potentially involved in lignin biodegradation. Therefore, it would be interesting to study the transcriptome during degradation in future studies.

Moreover, the strain tolerated and converted high concentrations (10 mM) of FA without growth inhibition. A comparable FA tolerance has only been described with an engineered *Pseudomonas putida* strain (Upadhyay et al. 2019). In comparison, FA concentrations above 5 mM had a growth-inhibiting effect on *Halomonas elongata* (Abdelkafi et al. 2006) and reduced the VA production yield of *Streptomyces sannanensis* (Ghosh et al. 2007); *Paenibacillus lactis* showed a very low conversion yield in the presence of 5 mM FA compared to 2.5 mM FA (Mishra et al. 2016).

An important criterion for biotechnological application is the bioconversion yield. In our study, the molar yield of VA produced from 5 mM FA was 88–89% and was marginally lower using 10 mM FA (85–86%) (Table 1). Remarkably, this high yield from pure FA was obtained at 10°C, 20°C, and 30°C. A comparable bioconversion molar yield from 10 mM FA has only been obtained with an engineered *P. putida* strain at 30°C (95%; Upadhyay et al. 2019) and – from only 5 mM FA – with *H. elongata* at 37°C (86%; Abdelkafi et al. 2006) and *Streptomyces halstedii* at 28°C (80%; Brunati et al. 2004). Other reported bioconversion molar yields are in the range of 60% (produced from 5 mM FA at 37°C by *Bacillus licheniformis*; Ashengroph et al. 2012), 38% (2.5 mM FA, 37°C, *Paenibacillus*; Mishra et al. 2016), or 11% (5 mM FA, 30°C, *Streptomyces setonii*; Muheim and Lerch 1999).

When considering the quite low amount of biomass produced during FA bioconversion by the strain used in this study, the performance becomes even more remarkable. Further studies to optimize the carbon balance should allow the optimal balance between growth rate, low substrate metabolism into biomass, and VA production without slowing down the conversion rate. This could be obtained by studying (i) the optimal media composition as previously reported (Ghosh et al. 2006; Mishra et al. 2016), (ii) in vitro conversion of FA to VA with cell extracts, and (iii) by increasing the amount of the substrate FA.

The reported bioconversion ability of the strain *P. aromaticivorans* AR20-38 to produce VA from FA is interesting from the viewpoint of application. FA (3-(4-hydroxy-3-methoxyphenyl)-2-propenoic acid) is very abundant in agricultural plants (by-products) as well as in softwood lignin and thus a natural renewable resource for the production of vanillin and VA (Ashengroph et al. 2012). There is a growing interest in exploiting microbial conversions of FA for the production of commercially valuable products, such as VA, vanillin, 4-vinyl guaiacol, and styrenes (Rosazza et al. 1995). VA (4-hydroxy-3-methoxy benzoic acid) production is especially interesting as this compound is used as the starting material in the chemical synthesis of vanillin (Rosazza et al. 1995) as well as polyesters; it is a potential food preservative and has been associated with a range of pharmacologic activities (Abdelkafi et al. 2006, Ghosh et al. 2007; Gitzinger et al. 2012). Besides FA, related abundant lignin monomers, such as p-coumaric acid, are attractive aromatic compounds of great value as precursors for other useful chemical products (Rosazza et al. 1995).

Studies on the biodegradation of lignin monomers have been conducted under mesophilic temperature conditions, i.e., at temperatures ranging from 28 to 37°C (Abdelkafi et al. 2006; Ghosh et al. 2007; Mishra et al. 2016; Ravi et al. 2017; Upadhyay et al. 2019). Our study is the first to report the biodegradation of lignin monomers (CA, HBA, FA, BA) and the bioconversion of FA to VA at lower temperatures. The ability of the studied strain for low-temperature growth and degradation can be attributed to its isolation source, an Alpine soil in a subalpine-continental climate with a mean annual air and soil temperature of 4.0°C and 4.3°C, respectively (França et al. 2016). The observed maintenance of a consistent degradation activity and a consistent conversion yield over a broad temperature range (10–30°C) is of biotechnological interest and is advantageous in environments that undergo thermal fluctuations. Such strains are useful for low-energy treatment of lignin and temperature-independent valuable product formation. Microbial cold adaptation includes a complex range of structural and functional adaptations at the level of all cellular constituents and offers multiple biotechnological applications (De Maayer et al. 2014; Margesin 2017; Collins and Margesin 2019).

In conclusion, the data obtained in this study demonstrate that *P. aromaticivorans* AR20-38 is characterized by a number of interesting capacities: (1) full degradation of a range of lignin monomers (5 mM CA, 5 mM HBA, 5 mM BA, and 5–10 mM FA at 10–30°C), (2) high bioconversion capacity for the stable production of VA from FA at 10–30°C: 88–89% (from 5 mM FA) to 85–86% (from 10 mM) molar yield at 10–30°C, and (3) tolerance to high amounts of FA without inhibition of growth and/or bioconversion. These features, obtained without further optimization, indicate the potential of the strain for biotechnological application. Its ability to utilize a number of other aromatic and polyaromatic hydrocarbons (phenol, catechol, naphthalene, phenanthrene) as sole carbon source (Berger et al. 2021) is an additional advantage. Moreover, biodegradation and bioconversion over a broad temperature range, including cold conditions, are of industrial relevance for low-energy treatments. Additional studies on gene expression would be of great interest to understand degradation mechanisms on a transcriptomic level.

## Data Availability

All data is presented in the manuscript, and the genome data can be found at the GenBank BioProject number PRJNA624061.
